# Proteomics-Based Insights Into the SARS-CoV-2–Mediated COVID-19 Pandemic: A Review of the First Year of Research

**DOI:** 10.1016/j.mcpro.2021.100103

**Published:** 2021-06-04

**Authors:** Jeremy L. Praissman, Lance Wells

**Affiliations:** Complex Carbohydrate Research Center, University of Georgia, Athens, Georgia, USA

**Keywords:** MS, glycosylation, SARS-CoV-2, COVID-19, review, ACE2, angiotensin-converting enzyme 2, CK2, casein kinase II, COVID-19, coronavirus disease 2019, DDA, data-dependent acquisition, nsp, nonstructural protein, PIKFyve, a FYVE finger–containing phosphoinositide kinase, PPI, protein–protein interaction, PRM, parallel reaction monitoring, PTM, post-translational modification, qPCR, quantitative PCR, SARS-CoV-2, severe acute respiratory syndrome coronavirus 2, TMPRSS2, transmembrane serine protease 2, TMT, tandem mass tag

## Abstract

In late 2019, a virus subsequently named severe acute respiratory syndrome coronavirus 2 (SARS-CoV-2) emerged in China and led to a worldwide pandemic of the disease termed coronavirus disease 2019. The global health threat posed by this pandemic led to an extremely rapid and robust mobilization of the scientific and medical communities as evidenced by the publication of more than 10,000 peer-reviewed articles and thousands of preprints in the first year of the pandemic alone. With the publication of the initial genome sequence of SARS-CoV-2, the proteomics community immediately joined this effort publishing, to date, more than 100 peer-reviewed proteomics studies and submitting many more preprints to preprint servers. In this review, we focus on peer-reviewed articles published on the proteome, glycoproteome, and glycome of SARS-CoV-2. At a basic level, proteomic studies provide valuable information on quantitative aspects of viral infection course; information on the identities, sites, and microheterogeneity of post-translational modifications; and, information on protein–protein interactions. At a biological systems level, these studies elucidate host cell and tissue responses, characterize antibodies and other immune system factors in infection, suggest biomarkers that may be useful for diagnosis and disease-course monitoring, and help in the development or repurposing of potential therapeutics. Here, we summarize results from selected early studies to provide a perspective on the current rapidly evolving literature.

## Highlights

• SARS-CoV-2, which caused the COVID-19 pandemic, depends on features of its proteome and host proteomes for transmissibility and virulence.
• Qualitative and quantitative proteomic assay development underlies attempts to further understand the virus.
• Protein–protein interaction and post-translational modification studies inform efforts to develop therapies.
• In sum, the range of proteomics technologies provides valuable information in the fight against COVID-19.

## Graphical Abstract

**Figure:**
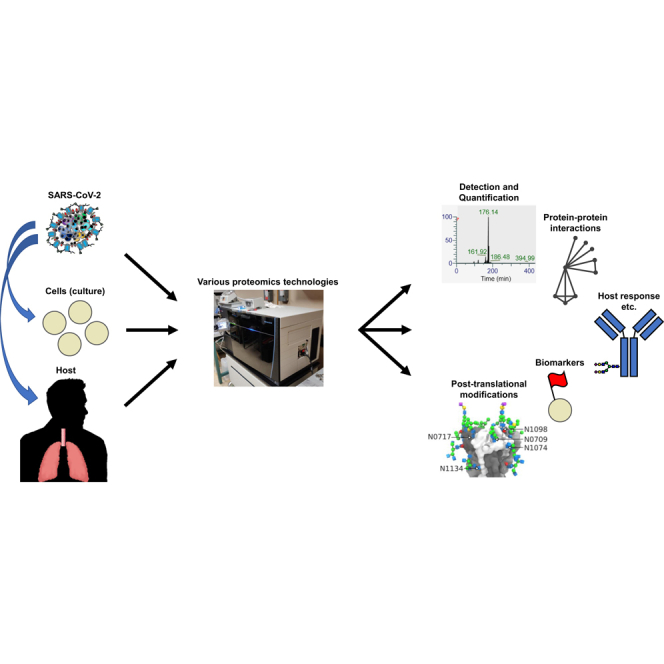

## In Brief

SARS-CoV-2, the betacoronavirus that caused the COVID-19 pandemic, became a major source of human disease and death in 2020. The fundamental constituents of a virus being its genome and proteome, characterizing the proteome is essential to understanding its biology. In this review article, we survey the proteomics literature from the first year of the COVID-19 pandemic, including protein–protein interaction studies, post-translational modification studies, and work using proteomics technologies to probe host response, which collectively inform efforts to ameliorate the pandemic.

Severe acute respiratory syndrome coronavirus 2 (SARS-CoV-2) is a betacoronavirus that began infecting people in 2019 with the index case identified as a hospitalized patient who initially became ill on December 1, 2019 (1). The genome of the then unknown etiological agent was rapidly sequenced and made available to other researchers in early January (2, 3, 4). The SARS-CoV-2 genome reported (29,903 bases, single-stranded RNA) was annotated as encoding 26 or more proteins and has high sequence similarity (~80% identity at the nucleotide level) to the extensively studied SARS-CoV-1 responsible for SARS outbreaks in 2002 and 2003 as well as to multiple animal coronaviruses (2, 4, 5, 6).

**Fig. 1:**
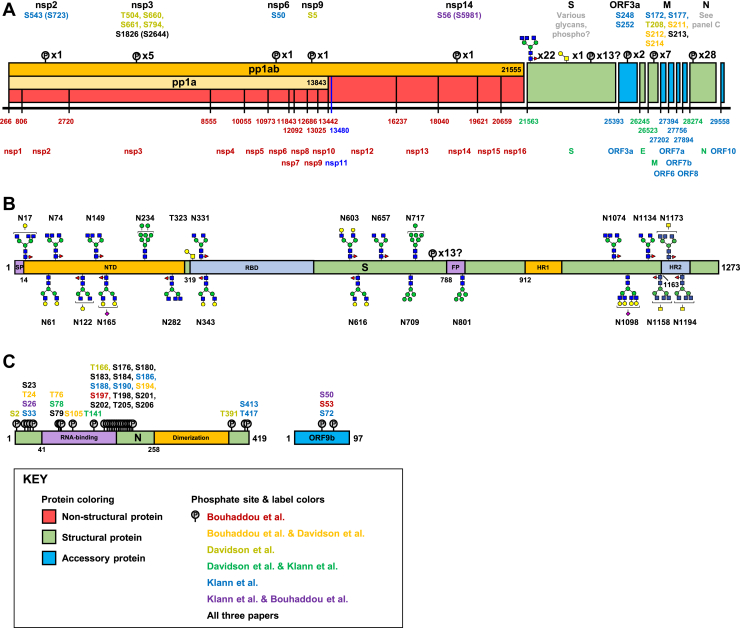
**The SARS-CoV-2 proteome and its post-translational modifications (PTMs).** The SARS-CoV-2 NCBI reference sequence proteome delineated along its genome (*A*). The 28 proteins annotated in the NCBI reference sequence are represented as *boxes* with the starting base corresponding to each protein in the genome listed later along with most protein names (pp1ab and pp1a are labeled inside *boxes*). Note that the nsp proteins are expressed as parts of large polyproteins (pp1ab and pp1a), which are subsequently cleaved by proteases contained in the polyproteins themselves. A summary of PTMs detected in proteomics studies is listed above each protein except for N and S, which are shown in detail in panels *B* and *C*. Numbers in parentheses indicate the residue number in pp1ab as given in the study by Klann *et al.* (102). The PTMs of S. A partial domain structure is shown for orientation with coloring for contrast and start residue numbers. The most abundant N-glycans from the most abundant Oxford class at each site are shown as reported by Zhao *et al.* (86). The class abundances at each site reported by Watanabe *et al.* (83) are similar although the protein they aonalyzed showed a small but clear tendency toward slightly less processed glycoforms. Articles have reported varying amounts of O-glycosylation on S almost exclusively at T323, occupancy generally ~10% or less. Note also that Davidson *et al.* (14) identified 13 sites of phosphorylation on S; however, most were not cytoplasmic. Secretory pathway kinases have been confirmed (*e.g.*, FAM20C), but it is not clear that these sites fit with known specificity determinants. The PTMs of N and ORF9b. Domain structure shown with coloring for contrast and start residue numbers. ORF9b is an alternative ORF in the N coding sequence that is not annotated in the NCBI reference sequence. FP, fusion peptide; HR1, heptad repeat 1; HR2, heptad repeat 2; NCBI, National Center for Biotechnology Information; nsp, nonstructural protein; RBD, receptor binding domain; SARS-CoV-2, severe acute respiratory syndrome coronavirus 2.

The SARS-CoV-2 National Center for Biotechnology Information reference genome was released shortly after publication of the initial genome sequences and contains annotations for 28 encoded proteins. Starting from the 5’-end, the annotated proteins consist of 16 nonstructural proteins (denoted as nsp1–nsp16), translated as components of large polyproteins and then separated by viral proteases, followed by structural proteins and additional ORFs at the 3’-end (Fig. 1). Putative functions of the encoded proteins were initially inferred by sequence homology to previously studied coronaviruses (2, 4, 5). The nsp proteins include two with protease functions essential for polyprotein processing—Mpro, also called 3CLpro (3C-like protease, nsp5) and PLpro (papain-like protease, nsp3)—as well as the viral replication–transcription complex subunits. Structural proteins encoded include spike (S), envelope (E), membrane (M), and nucleocapsid (N). A number of accessory proteins that have (partially) determined roles in host defense interference, intracellular trafficking, transcription, and replication in related coronaviruses are also encoded (4, 7, 8). In the context of proteomics, it is important to note that such homology-based annotations provide useful initial models but are necessarily subject to more definitive empirical characterization. This is particularly essential for most RNA viruses because of their comparatively compact genomes and the resulting multiform and multifunction nature of their encoded proteins driven by evolutionary constraints (9). Consequently, different groups have used a variety of marginally different annotations in their studies. Researchers have, therefore, continued to work on refining annotations of regulatory elements and encoded polypeptides that may not have been completely characterized by homology-based methods (10, 11, 12, 13, 14). For example, Finkel *et al.* (10) reported 23 unannotated ORFs in their study using ribosome profiling, and Davidson *et al.* (14) reported that 14% of the transcripts detected in their study do not code for a known ORF and subsequently identified peptides from these transcripts. To our knowledge, there is no current definitive database compiling this information, and interested readers are directed to the original research articles.

While the genome and preliminary information on the proteome of the SARS-CoV-2 itself were being defined, researchers also began determining the host cell proteins required for or facilitative of infection. Angiotensin-converting enzyme 2 (ACE2) was known to be the host cell surface receptor for several other coronaviruses, including SARS-CoV-1, and its identity as the host cell surface receptor for SARS-CoV-2 was quickly confirmed (15, 16, 17, 18). Mature membrane-bound ACE2 is a 788-amino acid single-pass type I membrane protein (~91 kDa without post-translational modifications [PTMs]) consisting of an N-terminal peptidase domain and a C-terminal collectrin-like domain that causes homodimerization at the cell surface and contains the transmembrane helix (19, 20, 21). Many viruses in addition utilize host cell proteases and other host cell machinery to enable and facilitate initial cell infection. The protease transmembrane serine protease 2 (TMPRSS2) was confirmed as a key factor in SARS-CoV-2 infection in one of the early articles confirming the identity of the cell surface receptor as ACE2 (18). Researchers have since continued to pursue potential alternative host cell surface receptors, related or alternative proteases (TMPRSS4, cathepsin B and L), and proteins involved in processes such as endosome maturation (a FYVE finger–containing phosphoinositide kinase [PIKFyve], two-pore channel 2) that are critical for infection (18, 22, 23, 24, 25, 26). However, many questions still remain regarding the role and behavior of host proteases and proteins during infection.

While experimental studies have continued, furthering understanding of the biology of SARS-CoV-2, important parallel efforts have focused on cataloging and increasing the accessibility of this information. Of particular note to the proteomics community are resources compiling genome sequences, annotations, protein–protein interactions (PPIs), PTMs, and proteomics datasets. Genome sequences for SARS-CoV-2 from which protein sequences may be derived are available from the Global Initiative on Sharing Avian Influenza Data (27), National Center for Biotechnology Information (28), European Molecular Biology Laboratory's European Bioinformatics Institute (29), and other organizations. The coronavirus disease 2019 (COVID-19) data portal (https://www.covid19dataportal.org/) from European Molecular Biology Laboratory's European Bioinformatics Institute maintains an updated curated collection of SARS-CoV-2 and host proteins and their relationships and information on pathways and from gene expression studies (29). A database specific for PPIs is available from the Biological General Repository for Interaction Datasets curation project (https://thebiogrid.org/project/3) and may be consulted for continuously updated information (30). Many proteomics datasets from relevant studies are available through ProteomeXchange and its subsidiary databases (http://www.proteomexchange.org/) (31). A number of additional databases contain proteomics data particularly useful for analyzing (or developing assays to analyze) host cell factors, for example, proteomicsDB (https://www.proteomicsdb.org/) (32), PAXdb (https://pax-db.org/) (33), the Clinical Proteomic Tumor Analysis Consortium Data Portal (https://cptac-data-portal.georgetown.edu/cptacPublic/) (34), Human Proteome Map (http://www.humanproteomemap.org/) (35), and The Human Protein Atlas (https://www.proteinatlas.org/) (36). Glycan information from various studies is compiled in GlyGen (https://www.glygen.org/) (37). In addition, glycan and other PTM information is available through PhosphoSitePlus (https://www.phosphosite.org/) (38).

In the first half of this article, we focus on the virus and its cell entry factors including the host cell receptor ACE2. The literature in this area may be further divided among (1) studies examining the basic qualitative behavior of viral and host cell entry factor peptides in mass spectrometric experiments; (2) quantitative proteomics studies that either detail viral protein expression over time or examine the distribution of host cell entry factors in human tissues and cells; and (3) studies of viral and ACE2 structure and PTMs. In the second half of this review, we provide an overview of studies focused on the proteomes of host (primarily human) cells and tissues and their responses and interactions with the SARS-CoV-2 virus. These studies encompass PPI mapping experiments, the quantitative proteomics of host cell protein expression during infection, determination of putative biomarkers, and characterization of immune system responses and SARS-CoV-2–directed antibodies during infection. Another review of SARS-CoV-2 proteomics was published during preparation of this article and may be of interest for further reading (39).

## Virus and Host Cell Entry Factor Studies

### Basic Qualitative Proteomics and Potential Clinical Diagnostics

A number of studies have been published containing information on the basic qualitative proteomics of the virus and the potential of proteomics technology–based assays for clinical diagnostics development. Important results include the determination of peptides suitable for targeted method development in LC–MS experiments in terms of level of detection and quantification, specificity and stability of amino acid sequences in reported genomes, and the presence or absence of PTMs. Testing of developed methods with relevant clinical samples has also been reported by several groups.

In an early study, Gouveia *et al.* (40) detected 101 (tryptic) peptides across six viral proteins (N, S, M, ORF1ab, ORF3a, and ORF8) from virus-infected Vero cells and further recommended 14 peptides for targeted assays (Table 1 in the article of Gouveia *et al.*). Zecha *et al.* (41) characterized both tryptic viral peptides (from cells or cell culture supernatant independently) and tryptic host cell peptides from four relevant cell line models (discussed further later in this review). Parallel reaction monitoring (PRM) assays for viral proteins were developed for 23 peptides with favorable properties in nanoflow PRM and 21 peptides in microflow PRM. For each peptide, the top six transitions are also detailed (supplemental Table S3 in the article by Zecha *et al.* (41), spectral libraries are available at Panorama Public (42)). Gouveia *et al.* and Zecha *et al.* recommended similar lists of peptides for targeted method development, sharing two peptides for M, five peptides for N, and one peptide for S. Additional articles have been published more recently and may be consulted for further reference (43, 44, 45) (Table 1).

**Table 1:** Selected peptides for SARS-CoV-2 detection and quantification reported in at least two publications

Accession	Protein	Peptide sequence	Theoretical unmodified precursor [M + H]+	Observed precursor *z*	Common mods	References
VME1_SARS2	M	EITVATSR	876.48	2	None	(40, 41)
		VAGDSGFAAYSR	1200.57	2	None	(40, 41)
NCAP_SARS2	N	ADETQALPQR	1128.57	2	Deamidation (NQ)	(40, 41, 43, 44, 50)
		AYNVTQAFGR	1126.57	2	Deamidation (NQ)	(40, 41)
		GFYAEGSR	886.41	2	None	(40, 41, 50)
		IGMEVTPSGTWLTYTGAIK	2025.04	2, 3	Oxidation (M)	(40, 41)
		NPANNAAIVLQLPQGTTLPK	2060.15	2, 3	Deamidation (NQ)	(40, 41)
SPIKE_SARS2	S	FQTLLALHR	1098.64	3	None	(40, 44)
		LQSLQTYVTQQLIR	1690.95	2, 3	None	(40, 41)

Host cell entry factor peptides have also been qualitatively characterized. Zecha *et al.* (41) developed PRM assays for ACE2 and TMPRSS2 (human and, partly nonshared, monkey) targeting 16 tryptic human ACE2 sequences and six tryptic human TMPRSS2 sequences (supplemental Table S1 in the article by Zecha *et al.*, spectral libraries are available at Panorama Public (42)). They found that ACE2 could be detected in all four of their model cell lines (ACE2-A549, an ACE2 overexpressor, Vero E6, Calu-3, and Caco-2) using PRM methods but only in two using a data-dependent acquisition (DDA)–based method. TMPRSS2 was only detectable in two of the cell lines tested (Calu-3 and Caco-2). Other known and potential viral entry factors including TMPRSS4, CTSB, cathepsin L, BSG (CD147), and FURIN variously appeared across cell lines in DDA data (41). In addition, numerous articles have reported analyses of *de novo* or publicly available proteomics datasets aimed at characterizing the cell, tissue, and bodily fluid distribution of relevant proteins and may be useful as further references (46, 47, 48, 49).

One longstanding goal in the proteomics field is the development of clinical diagnostics utilizing proteomics, and particularly MS-based, methods. This goal is of particular interest during a time of supply-chain disruptions and shortages of necessary reagents for PCR-based assays and other frequently used clinical laboratory methods. However, MS-based proteomics often suffers from sensitivity, specificity, and throughput issues. Zecha *et al.* (41) concluded that the PRM methods developed by their group were inadequate to serve as a reasonable clinical diagnostic platform. Furthermore, considering the literature more broadly (including preprints), they found wide variability among studies suggesting caution in attempting to apply these methods in a clinical setting. Additional studies may be consulted for further information on current progress in developing SARS-CoV-2 diagnostics from nasopharyngeal swabs, gargle solutions, other human samples, and simulated (mock) samples (from *in vitro*–derived mucus and inactivated virus) (43, 44, 45, 50, 51, 52, 53).

Additional studies not substantially focused on characterization of viral or host cell entry factor peptides but containing lists of detected peptides and further relevant information (often with deposited datasets available in various proteomics databases) have also been published (14, 51, 54, 55, 56, 57, 58).

### Quantitative Proteomics

Quantitative proteomics experiments involving SARS-CoV-2 and SARS-CoV-2 host cell entry factors generally fall into three categories: experiments quantifying virus (proteins), experiments examining the cell type and tissue distribution of host cell entry factors, and experiments quantifying changes in host cell (and other) factors during infection. Many of these experiments are natural extensions of the qualitative experiments outlined in the previous section.

**Fig. 2:**
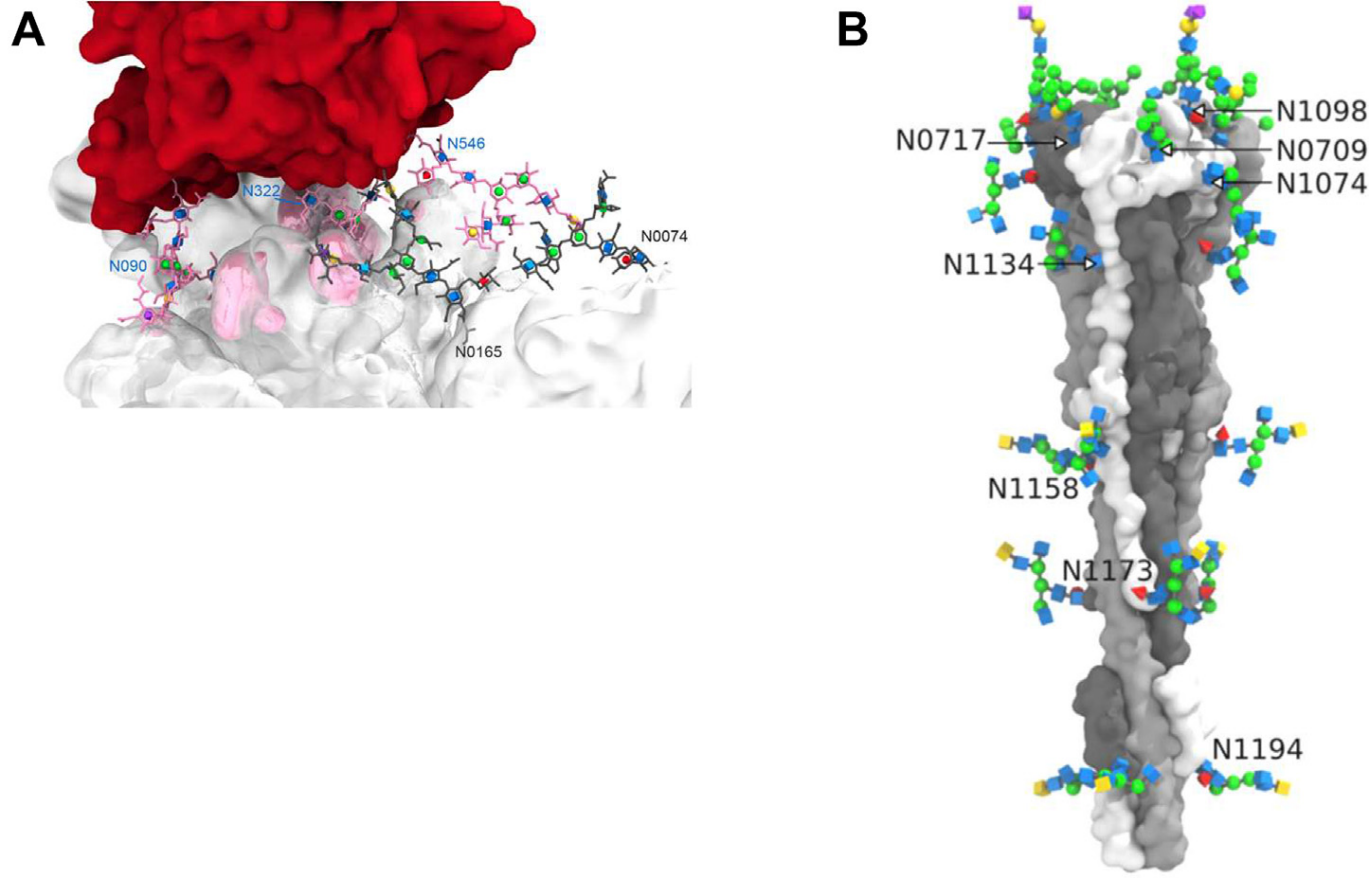
**Views of the SARS-CoV-2 spike protein and its glycosylation.** Images courtesy of Oliver C. Grant (unused graphics from Zhao *et al.* (86)). Protein models courtesy of Professor Bing Chen. *A*, the interface of SARS-CoV-2 S (*white*) bound to ACE2 (*red*) showing glycans involved in glycan–peptide and glycan–glycan interactions. *B*, the postfusion structure of SARS-CoV-2 S showing its distinctive columnar structure and regular spacing of N-glycans. ACE2, angiotensin-converting enzyme 2; SARS-CoV-2, severe acute respiratory syndrome coronavirus 2.

Quantification of SARS-CoV-2 proteins has been carried out to understand the kinetics of viral infection, examine the effect of administration of potential therapeutics, and assess viral abundance in infected patients. Grenga *et al.* (58) characterized viral kinetics by assaying viral protein expression at several time points (day 1, 2, 3, 4, or 7) for cultures infected either at multiplicity of infection 0.01 or 0.001 on day 0. These results were compared with quantitative PCR (qPCR) measurements of viral RNA and found to be consistent validating their methods in this context (Grenga *et al.*; Fig. 2). Appelberg *et al.* (59) also looked at viral RNA levels by qPCR as compared with viral protein abundance over time (24, 48, or 72 h after infection) from SARS-CoV-2–infected Huh7 cells using a tandem mass tag (TMT)–based method (TMT-MS) and found similar trend agreement between qPCR results and their developed TMT-MS method (Appelberg *et al.*; Fig. 1). Gordon *et al.* (56) quantified virus protein expression at 8 h after infection in cell culture in relation to treatment (singly) with three potential therapeutic compounds previously identified in their study through other methods (two ligands of sigma-1 and sigma-1 receptors and one protein biogenesis inhibitor), confirming the effectiveness of these compounds in putatively disrupting viral replication. Zecha *et al.* applied their PRM-based methods to quantify viral proteins in patient samples, although their assays were designed for repeatability rather than for accurate and precise quantification. For samples in which SARS-CoV-2 peptides were detected, peptide intensities were generally in good correspondence with PCR results. However, the PRM assay had a prohibitive rate of false negatives in patient samples (43 of 54 or approximately 80% false negative), a difficulty encountered with MS-based assays in general (41). Gouveia *et al.* further developed and tested a method based on their first article (on SARS-CoV-2 peptide analytical characteristics) establishing a lower limit of detection and concluding that two tryptic peptides from the nucleocapsid protein provide the best basis for a DDA (with inclusion list) reversed-phase LC–MS/MS-based diagnostic platform of the type described. However, these experiments similarly achieved a low rate of detection with diagnosed patient samples (two of nine patients or ~22% from a PCR validated cohort) (50). Relevant peptides from these studies are summarized in Table 1.

Several studies have been published to date examining the tissue and cell-type distribution of host cell entry factors. One of the classic studies is by Hamming *et al.* (60), published in 2004 shortly after the original SARS outbreaks and still a very relevant resource to consider newer research against, although not MS based. In more recent research, Zecha *et al.* (41) were able to relatively quantify ACE2 in their four cell line models, ACE2-A549, Vero E6, Calu-3, and Caco-2, using a PRM-based method they developed (discussed in more detail previously), finding expression to be more than 1000 times lower in Calu-3 and Caco-2 cells compared with ACE2-A549 cells. TMPRSS2 and other factors involved or putatively involved in host cell entry also varied widely in expression (Fig. 1, supplemental Fig. S1*C*, and supplemental Table S1 in the article by Zecha *et al.*). Hikmet *et al.* (46) and Aguiar *et al.* (61) carried out immunohistochemical analysis of tissue and protein profiling by Western blot and from two publicly available MS datasets (one in common to both articles, Kim *et al.*, 2014 (35)). Both articles extensively analyzed tissue and cell distribution finding a similar pattern of ACE2 expression (high abundance in kidney and testis, lower abundance in gallbladder, and so on; Figs. 4–6 in the article by Aguiar *et al.* (61), Fig. 6 in the article by Hikmet *et al.* (46)). Aguiar *et al.* (61) also profiled TMPRSS2, BSG (CD147), and HSPA5 in detail (Figs. 4–6 in the article by Aguiar *et al.*). Wang *et al.* (47) carried out a similar analysis entirely using previously published data and generating similar results (Fig. 2 in the article by Wang *et al.*). Finally, Stanley *et al.* looked specifically at reproductive cells using data from the Human Protein Atlas (36) and the Human Proteome Map (35) and concluded that reproductive consequences of SARS-CoV-2 infection are low given a lack of detectable coexpression of ACE2 and TMPRSS2 at the protein level (49). One of the most interesting results from these and prior studies, considering the primary respiratory route of viral transmission, has been the difficulty of detecting ACE2 and TMPRSS2 in upper airway samples (61) and other lung tissue samples (46, 47, 61). Researchers have typically explained this by suggesting that alternative entry factors may exist, noting that ACE2 expression appears to be restricted to a subset of (generally) epithelial cells, and by noting that interferon can upregulate ACE2 expression once infection is established (60, 61, 62). Among other tissues notable in pathogenesis and symptom presentation, ACE2 and TMPRSS2 were codetected in multiple intestinal samples (46, 47, 61), and intestine has long been known to be particularly enriched in ACE2 expression (60).

Studies have also examined changes in host cell entry factor abundance during infection. Understanding the role of ACE2 modulation during infection is clinically significant because ACE2 is both the viral receptor as well as a lung protective factor (notably in SARS-CoV-1 infection) (18, 62, 63, 64, 65, 66, 67, 68). However, characterizing this modulation has proven challenging as ACE2 (cell surface) levels are variously upregulated or downregulated by different factors in infection including interferon signaling and proteases (*e.g.*, ADAM17) (69, 70). In SARS-CoV-2 infection, Bojkova *et al.* (and Bock *et al.* (71) based on the same dataset) have now reported reduction of ACE2 abundance in the Caco-2 cell line (a human colon carcinoma line) (57). In contrast to these data, however, Zecha *et al.* (41) did not find ACE2 decrease in their cell line models (ACE2-A549, Vero E6, Calu-3, and notably also Caco-2), although they found a decrease in abundance of cathepsin L over time. Continued work will further refine our understanding of host cell entry factor changes during the course of infection.

### PTMs

Coronavirus proteins, receptors, and other relevant host cell proteins are often post-translationally modified (72, 73). Review of the SARS-CoV-2 proteome literature revealed studies of glycosylation, phosphorylation, and at least one report detailing (lysine, arginine, and glutamic acid) methylation and proline oxidation on SARS-CoV-2 S and human ACE2 produced in insect cells (74). The methylation results are somewhat surprising since most methyltransferases are localized to the nucleus although there are reports of aspartic and glutamic acid methylation in the secretory pathway (75). No studies have yet confirmed other modifications typically observed with coronaviruses such as ADP ribosylation, sumoylation, palmitoylation, or ubiquitination although sites have been predicted by bioinformatics (73, 76, 77), and relevant protein interactions for such modifications have been demonstrated (56, 71, 78).

#### Glycoproteomics and Glycomics

Several SARS-CoV-2 proteins (S, M, E, and certain “orf” proteins such as Orf8 (79, 80)—a viroporin—and likely Orf7 (81), as with related coronaviruses), as well as host cell factors important in infection, transit the secretory pathway during expression and thus may be glycosylated by secretory pathway glycosyltransferases. The “S” protein (also called “spike” or “surface glycoprotein”) assembles as homotrimers and coats SARS-CoV-2 virions (16, 17). The primary binding partner of spike for host cell entry, ACE2, is also a glycoprotein (15, 18, 82). Representing natural targets for both antibodies and inhibitors, there has been substantial interest in both these proteins and their protein-linked carbohydrate moieties that may shield or otherwise alter PPIs and protein accessibility. In reviewing glycoproteomic and glycomic studies (particularly preprints), it is important to note that recombinant protein design may lead to non-native modifications (*e.g.*, reports of secretory pathway glycosylation of N).

To date, there have been six glycoproteomics and glycomics studies published on the carbohydrates covalently attached to SARS-CoV-2 spike, two of which also characterized the glycosylation of ACE2 (74, 83, 84, 85, 86, 87). All results published to date confirm that the spike protein is predominantly modified by N-glycans (at 22 sites) and that there may be varying amounts of O-glycans present at one site (T323). Distinguishing parameters of primary importance among these results are the cell model, recombinant protein design, and purification strategy used. Two of the studies utilized experimental designs (human cell line expression and trimer purification) that have previously been widely shown with viruses in general to produce proteins very close in character to those derived from actual viral infections (83, 86). These studies, by Watanabe *et al.* (83) and Zhao *et al.* (86), were in substantial agreement regarding the identities of glycans present and the occupancy of each glycosite and in addition demonstrate the importance of multiple protease digestion and the use of different types of fragmentation activation for comprehensive glycan and glycosite characterization. Zhao *et al.* (86) were also one of only two studies to date in which glycomics was carried out to refine the topologies of the glycans present (87). Other studies cited previously either utilized proteins not produced in human cell lines (74, 85) or protein other than full-length trimer purified spike (84, 87), raising additional questions as to the biological relevance of the glycosylation results obtained with respect to actual SARS-CoV-2 virions in human hosts. The apparent resulting differences provide valuable information to researchers considering antibody or vaccine candidate production in nonhuman cell lines or using nontrimer purified protein. Finally, it is worth noting that SARS-CoV-2 glycosylation is significantly more host like than the glycosylation found on many other viruses such as HIV when considering N-glycan processing and density, although high mannose glycans still occur with greater prevalence than on most host proteins (83, 86, 88).

The two recent studies characterizing the glycosylation of ACE2 form a subset of the SARS-CoV-2 spike articles (74, 86) and provide a much more complete picture than earlier articles (89, 90, 91, 92). In particular, Zhao *et al.* (86) carried out comprehensive glycomics-informed glycoproteomic analysis on a purified soluble version of the protein. ACE2 has seven “canonical” N-glycosylation sequons, six of which were included in the expression construct used in the article and analyzed in depth. The N-glycosylation of ACE2 was found to be broadly similar to that seen with other human proteins that traffic through the secretory pathway. Only a small amount of O-glycosylation was detected. This detailed analysis of glycosylation also allowed the authors to carry out molecular dynamics simulations of SARS-CoV-2 S glycoprotein bound to ACE2 glycoprotein suggesting that several N-linked glycans on each protein are intimately involved in glycoprotein–glycoprotein interaction (Fig. 2). In addition, a model of the postfusion SARS-CoV-2 S glycoprotein was developed showing its distinctive columnar structure and even N-glycan spacing (Fig. 2). A number of other articles containing molecular dynamics simulation results and molecular modeling work have been published based on different glycoproteomics studies and are valuable additional references (93, 94, 95, 96, 97, 98, 99, 100). Sun *et al.* (74) reported very similar results on the N-glycosylation of ACE2. Although they were unable to detect N-glycans at N053 and N322, by using a construct including the N-glycosylation site N690, Sun *et al.* (74) were able to characterize glycosylation at this seventh site in contrast with Zhao *et al.* (86). In total, the glycosylation patterns of the SARS-CoV-2 spike protein and its host cell surface receptor have been characterized in detail by multiple groups using different biological models providing important information for future research and particularly informing modeling that may be crucial in understanding and addressing the emergence of potential vaccine and antibody escape variants.

#### Phosphorylation

**Fig. 3:**
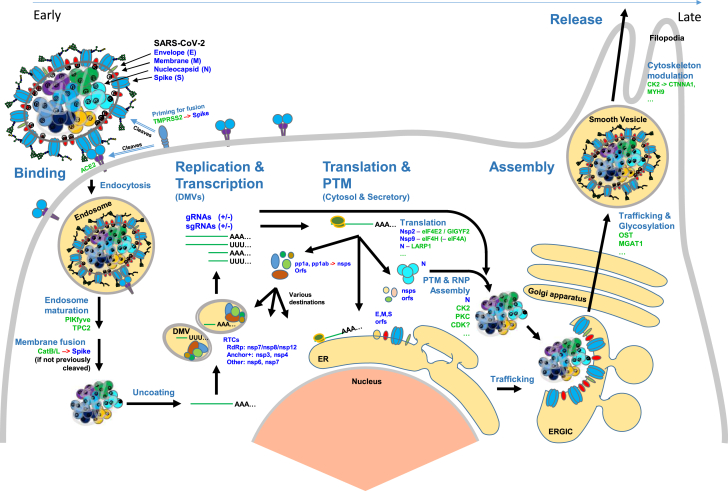
**The SARS-CoV-2 viral life cycle and selected host proteins involved.** The viral life cycle is displayed proceeding from host cell entry through new virion synthesis, packaging, and export. Host cell proteins are labeled in *green*, and SARS-CoV-2 proteins are labeled in *blue*. *Red arrows* (→) indicate protease cleavage. The representation of virus shows ribonucleoproteins (RNPs) (consisting of five dimers of N) in the tetrahedral geometry recently reported (Yao *et al.* (99)). This article reported an average of 26 ± 15 copies of prefusion S per virion and 26 ± RNPs per virion. The life cycle in a given cell begins with host cell entry mediated by ACE2 (the receptor), TMPRSS2 (or alternatively CatB/L—CSTB/CTSL—fusion priming enzymes), and proceeds with trafficking through endosomes. Endosomal maturation required for viral–host–cell membrane fusion involves the proteins PIKfyve and TPC2. After fusion and uncoating of the viral RNA, the replication-transcription complex is expressed, and new viral genomic RNAs (gRNAs, + and − sense) and subgenomic RNAs (sgRNAs, + and − sense) are produced. The translation of viral proteins and modulation of host protein translation is affected by protein–protein interactions (Nsp2-eIFE2/GIGYF2, Nsp9-eIF4H, and N-LARP1 are shown) and signaling. New virion structural protein N is phosphorylated (CK2, PKC, and CDK), forms RNPs, winds gRNAs, and collects at the ERGIC membrane for envelopment. Viral proteins E, M, and S traffic through the secretory pathway for further processing including addition of glycans. Filopodia formation is enhanced (proposed to be CK2 driven by Bouhaddou *et al.* (101)) and may improve transmission of egressing virus between cells. ACE2, angiotensin-converting enzyme 2; CTSL, cathepsin L; ERGIC, endoplasmic reticulum golgi intermediate compartment; SARS-CoV-2, severe acute respiratory syndrome coronavirus 2; TMPRSS2, transmembrane serine protease 2.

Currently, three published studies have specifically examined the phosphorylation of SARS-CoV-2 proteins (Fig. 1) and host cell entry factors (14, 101, 102). Davidson *et al*. (14) used TiO_2_ followed by ferric nitrilotriacetate phosphopeptide enrichment on in-gel trypsin-digested peptides from whole cell lysate enabling the mapping of 44 phosphorylation sites among five viral proteins (Figs. 3–5 and Table 4 in the article by Davidson *et al.*: nsp3, nsp9, M, N, and S—nsp12 and ORF3a also produced phosphorylated peptides, but sites could not be confidently assigned) in a typical DDA LC–MS/MS higher-energy collisional dissociation experiment (14). The report of phosphorylation sites distributed across spike is surprising, as noted by Davidson *et al.* in their article, given its secretory pathway expression, and it will be interesting to see if future studies confirm this result. Bouhaddou *et al.* (101) comprehensively examined phosphorylation of both viral proteins and host proteins, enriching from cell lysate tryptic peptides *via* iron affinity and analyzing *via* LC–MS/MS (DDA and data-independent acquisition) with higher-energy collisional dissociation. Using this approach, they identified 25 phosphorylation sites (omitting results from Davidson *et al.* (14) that they also presented) on SARS-CoV-2 viral proteins (Fig. 2 and supplemental Table S2 in the article by Bouhaddou *et al.* (101): on nsp3, nsp14, orf9b, M, and N). The phosphorylation of N has since been shown to be functionally important in nucleocapsid assembly and viral replication and transcription (103, 104, 105) as was previously observed with other coronaviruses. Several sites in the C-terminal tail of M are noted to be present in other viruses suggesting potential functional importance although it does not appear that this has been functionally verified for SARS-CoV-2 yet. Among host cell entry factors, PIKfyve and cathepsin L are phosphorylated (Table S1 in the article by Bouhaddou *et al.* (101)), and regulation of the phosphorylation of PIKfyve was further confirmed to be involved in SARS-CoV-2 infection (101). Klann *et al.* (102) used ferric nitrilotriacetate enrichment after in-solution digest and TMT labeling of proteins from whole cell lysate finding 33 sites on six viral proteins (Fig. 1, *E*–*J* in the study by Klann *et al.* (102): pp1ab, nsp6, ORF3a, ORF9b, M, and N). Additional datasets available in the ProteomeXchange (31) contain various sets of relevant phosphorylation hits and may be found by searching this database.

## Host Cell Proteomics

### PPI

PPIs play a primary role in the life cycle of animal viruses, from attachment to cells through endosomal compartment escape (for most viruses) and ultimately reorganization of cellular machinery to support viral reproduction and diminish host defense (106, 107). Consequently, enumerating host–virus PPIs is crucial to understanding the biology of viruses and developing a starting point for investigation of potential therapeutics. To date, several comprehensive studies of SARS-CoV-2 host–virus PPIs using proteomics technologies have been published, setting the stage for additional studies expanding on this work—studies that may definitively validate or invalidate proposed interactions. An extremely useful resource for SARS-CoV-2 PPIs was brought to our attention during review of this article, and interested readers are directed to it for a more comprehensive up-to-date view of published interactions: https://thebiogrid.org/project/3 (30). The interactions reported in this database and in the research cited later may also be considered in the context of more recently published functional genomics studies for additional perspective (25, 26, 108, 109, 110).

A comprehensive interactome was published by Gordon *et al.* who carried out affinity purification, using transfected viral proteins (human embryonic kidney 293 cells) as baits against human cell proteins in the cells used for viral protein expression, followed by tryptic digestion and LC–MS/MS (56). Through this workflow, they were able to map 332 high-confident PPIs and identify 66 druggable human proteins concluding, through additional experiments with compounds targeting interactors, that inhibitors of mRNA translation and regulators of sigma-1 and sigma-2 receptors show potential for SARS-CoV-2 treatment. Noteworthy PPIs involve factors in host mRNA nuclear export and overall mRNA regulation/translation, phosphorylation, secretory pathway targeting of proteins, and protein degradation (Fig. 3, Table 2, and supplemental Table S1). This group has since published a newer study comparing interactors across related coronaviruses SARS-CoV-1 and Middle East respiratory syndrome-CoV and validating or further validating the clinical relevance of three host factor interactors reported in their original study (Tom70 or TOMM70, ILR17RA, and SigmaR1) (111). Li *et al.* (78) more recently published another comprehensive interactome using similar affinity-purification LC–MS/MS methods and found 45 targets shared with the article by Gordon *et al.* (56) (~16% of their interactome, including, *e.g.*, SigmaR1) while uncovering many unreported PPIs. In particular, Li *et al.* found many immune system–related interactions (78) (Table 2 and supplemental Table S1).

**Table 2:** Selected host proteins in infection

Primary process	Gene/complex/family	Protein name	PPI?	Abundance?	Phosphorylation?	Act.?	Function in infection (known and/or hypothesized)	Cell location	Selected SARS-CoV-2 proteomics references
Host cell entry	ACE2	Angiotensin-converting enzyme 2	S	+/−			Virus receptor	PM	(41, 46, 47, 48, 49, 57, 61, 74, 86)
	TMPRSS2	Transmembrane protease serine 2	S				Cleaves S (“priming”), especially at S2' site	PM	(41, 48, 49, 61)
	CTSB	Cathepsin B	S?				Cleaves S, alternative to TMPRSS2	EN	(41, 46, 78, 112)
	CTSL	Cathepsin L	S				Cleaves S, alternative to TMPRSS2	EN	(41, 46, 49, 61, 112)
Endosomal release	PIKFYVE	1-phosphatidylinositol 3-phosphate 5-kinase					Endosome maturation, with TPC2	EN	(101)
Protein expression	NUP98	Nuclear pore complex protein Nup98	Orf6		+		Prevent host nuclear mRNA export	NM	(56, 101)
	LARP1	La-related protein 1	N		-		Prioritize virus protein expression	CP, NU	(56, 101, 112)
	UPF1	Regulator of nonsense transcripts 1	N				Binding by N represses NMD?	CP, NU	(56, 112)
	EIF4H	Eukaryotic translation initiation factor 4H	Nsp9				Cap-dependent mRNA translation	PN	(56)
	EIF4E2	Eukaryotic translation initiation factor 4E type 2	Nsp2				Represses cap-dependent translation	CP	(56, 112)
	Sec61 complex	SEC61 channel-forming translocon complex	Nsp8				Protein entry into endoplasmic reticulum	CP, ERM	(56, 57)
	BRD4	Bromodomain-containing protein 4	E	+			Interference with antiviral response?	NU	(48, 56)
Protein processing	FURIN	Furin	S				Cleaves S (“priming”), especially at S1/S2 site	Golgi	
Protein degradation	CUL2	Cullin 2	Orf10	+			Increase degradation of restriction factors?	CP, NU	(56, 59)
Cell signaling	CDK	Cyclin-dependent kinase				−	Cell cycle arrest, S/G2	NU, MT, CP	(101)
	MAPK	Mitogen-activated protein kinase		+		+	Viral replication+, stress response	NU, CP, MT	(59, 101, 102, 122)
	AKT	RAC-alpha serine/threonine-protein kinase		+	+	−/+	Viral replication+, cell proliferation & apoptosis regulation	NU, CP (PM)	(59, 101)
Cell structure	PHB complex	Prohibitin complex	nsp2				Signaling interference, mitochondrial antiviral signaling, apoptosis−	MT, NU, CP, PM	(56, 112, 129)
	CK2 complex	Casein kinase II	N			+	Cytoskeleton changes, filopodia+	CP, NU	(56, 101, 102)
Stress, immunity	HSPA5	Endoplasmic reticulum chaperone BiP					Unfolded protein response, virus receptor?	CP, PM	(61)
	NKRF	NF-kappaB–repressing factor	(nsp10)				IL-8 induction	NO, NU, CP	(78)
	CFB	Complement factor B		−/+		+	Alternative complement pathway factor	Secreted	(120, 121, 122)
	CFD	Complement factor D					Activates complement-dependent killing	Secreted	(120, 123)
	CFI	Complement factor I		+		+	Prevented from modulating complement	Secreted	(120, 123)
	CFH	Complement factor H		+/−		+	Prevented from modulating complement	Secreted	(120, 122, 123)

Abbreviations: Cell location—CP, cytoplasm; EN, endosome; ER, endoplasmic reticulum; ERM, ER membrane; LY, lysosome; MT, mitochondria; NM, nuclear membrane; NO, nucleolus; NU, nucleus; PM, plasma membrane; PN, perinuclear; other—HS, heparan sulfate; MAVS, mitochondrial antiviral signaling; NMD, nonsense mediated decay.

See supplemental Table S1 for more information on these proteins, complexes, and families. NKFR = nsp10 PPI may not be direct.

Two studies have used interactome data previously published on other coronaviruses to predict the SARS-CoV-2 interactome, in one case thoroughly integrating this analysis with the data by Gordon *et al.* (56). Perrin-Cocon *et al.* (112) integrated the Gordon *et al.* (56) interactome with coronavirus–host interactions from literature on 12 other coronaviruses. Many of the interactions found were already widely reported although several previously relatively overlooked (for SARS-CoV-2) interactions were noted including with protein translation repressors, mitochondrial homeostasis regulators, and an S-adenosylmethionine synthase crucial to the DNA methylation pathway (Table 2 and supplemental Table S1) (112). Messina *et al.* (113) carried out a more restricted analysis based only on the spike protein and without integrating the findings of Gordon *et al.* (56), highlighting several noteworthy pathways (113) (Table 2 and supplemental Table S1). It is important to note that functional genomic screens that are now available have not directly confirmed the biological importance of many of these interactions, in some cases, potentially because of their design and limitations.

Several studies have integrated results from Gordon *et al.* (56) with other types of host proteome data. Feng *et al.* (48) integrated the interactome with proteomics datasets from human lung, colon, kidney, liver, and heart, to piece together a more tissue-centric view of the results from Gordon *et al.* (56). They also extended this analysis with differential quantitative “infectome” data from Bojkova *et al.* (57) (discussed in detail in the next section) carrying out further PPI network analysis and identifying critical “hubs” and hub proteins, grouped by tissue, and involved in mRNA processing (HNRNPC, SRSF1, and HNRNPA3), gene expression (BRD4), the tumor necrosis factor signaling cascade (RIPK1), and the olfactory signaling pathway (REEP5) among others (48). Zecha *et al.* (41) integrated the results from Gordon *et al.* (56) with their own quantitative data on the host proteomes of four relevant cell lines (human ACE2-A549, Caco-2, Calu-3, and African green monkey Vero E6) finding 56 interacting host proteins differing quantitatively during infection of Vero E6 cells (Zecha *et al.*; Figure 4*C*, supplemental Fig. S8*D*) (41). Appelberg *et al.* (59) also analyzed interactions in Gordon *et al.* (56) in terms of quantitative transcriptomic and proteomic data (TMT based) that they generated (Huh7 cells) (59). Finally, Bouhaddou *et al.* (101) integrated information from the Gordon *et al.* (56) interactome with quantitative proteomics and phosphoproteomics as discussed in more detail in next section.

### Host Cell and Tissue Proteome Changes in Infection

Related to direct PPIs is the set of proteins regulated or otherwise modulated during infection. This set includes many proteins involved in cell survival and the cell cycle, signaling, host defense (often directly inhibited by pathogenic viruses), cell trafficking, and other critical cellular and host processes (114, 115). In contrast with studies of PPIs using techniques like affinity purification, differential quantitative proteomics is subject to several additional confounding factors. First—viral protein expression increases in particularly permissive host cells dramatically over the course of even relatively short time intervals (8 h–2 days), which can negatively impact sensitivity and dynamic range in addition to making accurate comparative quantification challenging (58, 101). Second—in cell culture, different populations of cells will exist concurrently at any given time point (uninfected cells through to apoptotic, necrotic, and fully lysed cells). Third—in patient samples, quantified proteins must be compared against some standard usually chosen to be “uninfected controls.” This raises questions about how closely the proteomes of small sets of people (in the studies carried out to date) match under ordinary conditions. In this section, we will discuss articles on differential quantitative proteomics of host cell lines in SARS-CoV-2 infection and briefly mention extensions of those studies. We will follow this with discussions of differential quantitative proteomics (and potential biomarkers) from patient samples.

Beginning with the publication of Bojkova *et al.* (57), initially available as a preprint in March 2020, seven articles have reported host primary cell or host cell line quantitative differential proteomics in the context of SARS-CoV-2 infection. Six of these articles carried out extensive differential expression, network, pathway, and gene ontology analysis (57, 58, 59, 78, 101, 102) (Bouhaddou *et al.* (101) and Klann *et al.* (102) primarily in terms of the phosphoproteome), whereas one examined differential expression in a more cursory fashion (41). Studies differed in cells or cell line models used (patient peripheral blood mononuclear cells (78), Caco-2 from human colon carcinoma (57, 102), Vero E6 from African green monkey kidney epithelial cells (58, 101), and Huh7 from human hepatocyte carcinoma (59)), time points examined, and analytical techniques and technologies. Three groups utilized TMT labeling for quantification (41, 59, 78), one group used multiplexed enhanced protein dynamics proteomics based on pulsed stable isotope labeling by amino acids in cell culture and TMT (57) for their first article and TMT alone for their follow-up article (102), and the other two groups used forms of label-free quantification (58, 101). Pathways identified in common among various articles include spliceosomal (57, 58, 101, 102), hypoxia-inducible factor 1 signaling (41, 57, 59), innate immune system (41, 59, 78, 101), and pathways involved in carbon metabolism (57, 58, 59, 78, 102). Many of the host proteins shown to directly interact with viral proteins do not appear centrally in these results presumably since interaction is distinct from regulation. However, for purposes of comparison, HMOX1 (heme degradation) (41, 56, 59, 78), RIPK1 (NF-kappaB, tumor necrosis factor signaling) (56, 59), CUL2 and RBX1 (ubiquitination) (57, 59), and La-related protein 1 (an mechanistic target of rapamycin–regulated translation repressor) (56, 59, 101) are some proteins shared between PPI studies and these studies (Fig. 3, Table 2, and supplemental Table S1). Also of interest, certain pathways such as complement and innate immune activation were detected as differentially regulated only at low multiplicity of infections and/or in certain cell lines (41, 59).

Two of the differential quantitative proteomics articles examined the phosphoproteome in depth and found significant phosphoproteome changes consistent with the central role of phosphorylation in cell signal transduction. Bouhaddou *et al.* (101) compared the proteome and phosphoproteome of infected cells at six time points (0, 2, 4, 8, 12, or 24 h) and noninfected cells at two time points (0 or 24 h). This analysis revealed changes in phosphorylation of proteins involved in RNA processing (including La-related protein 1 and ribosomal RNA processing 1), nuclear export (NUP98; Table 2, supplemental Table S1), cytoskeleton organization and filopodia formation (VIM, STMN1, CTNNA1, MYH9; Fig. 3, Table 2, and supplemental Table S1—see casein kinase II [CK2]), the p38/mitogen-activated protein kinase pathway (NELFE, HSPB1, and STAT1), and cell cycle arrest (cyclin-dependent kinase 2; Fig. 3, Table 2, and supplemental Table S1). Based on the amino acid sequences of mapped sites, kinases and kinase families involved were also predicted (CK2, cyclin-dependent kinase, PKC, mitogen-activated protein kinase 12, CAMK2G, and AKT1/2 among others; Fig. 3, Table 2, and supplemental Table S1). In addition, Bouhaddou *et al.* were able to classify phosphoproteome changes into sets based on their appearance throughout the viral life cycle (early, replication, and egress). Finally, Bouhaddou *et al.* (101) also analyzed their results in the context of the Gordon *et al.* interactome (56) generating additional hypotheses regarding the mechanisms of a subset of the regulation they observed (*e.g.*, allosteric regulation of CK2 by N). Klann *et al.* (102) carried out similar analyses finding significant regulation of proteins involved in numerous pathways, including receptor signaling, endocytosis, the cell cycle, translation initiation, and splicing. Although there is some overlap between proteins revealed by these two studies, there are clear differences in the set of proteins and pathways reported that will not be covered further in this review.

Several additional articles consisting of reanalyses or extensions of data from the articles discussed previously have also been published. Feng *et al.* (48), as discussed earlier, reanalyzed and extended the data from Bojkova *et al.* (57) and Gordon *et al.* (56) to the tissue level. Bock *et al.* (71) reanalyzed the data from Bojkova *et al.* (57) using different methods highlighting, for example, the complement cascade in contrast with the original analysis. Many additional articles utilizing these differential host proteomics datasets and results have also been published (10, 102, 116, 117).

### Host Biofluid Proteome Changes, Biomarkers, and Immune Response

A number of extensive reviews have recently been published containing information on host biofluid proteome changes, potential biomarkers, and immune system response. Therefore, we will not cover the primary literature in these areas in detail here but will briefly summarize several reviews pointing the reader to them for more extensive discussion.

One recent review focused substantially on proteomics was published by Whetton *et al.* Whetton *et al.* (118) discussed the complexity of COVID-19, covered relevant proteomics techniques, compiled a table of selected publications detailing proteome changes in disease, presented network analyses of coronavirus- and SARS-related PubMed abstracts (in terms of cytokines, cells, and connected diseases), and concluded that future pandemic response would greatly benefit from more integrated OMICS and informatics pipelines. Ultimately, in terms of characterized proteome changes, they highlight the widely reported cytokine storm profile present in patients. They also point out markers associated with disease severity, including IL-6, troponin I, and B-type natriuretic peptide. Of particular interest, in light of a recent report on the importance of the alternative pathway of the complement system in COVID-19 disease pathogenesis (119), Whetton *et al.* (118) called attention to the appearance of alternative pathway–relevant proteins in a couple of proteomics articles (120, 121). Additional proteomics articles examining complement system proteins in SARS-CoV-2 infection have since been published (122, 123) (see also Table 2 and supplemental Table S1). Another recent review of note but less focused on proteomics in isolation (among others (124, 125)), focusing rather on immune response and immunological pathways is by Vabret *et al.* and expands on the immunology and biochemistry of much of this information (126). This review also includes information on current clinical trials and therapeutic options. A number of other articles with a proteomic character, including many previously cited in our review (several only in tables), also point to various immune pathways and biofluid proteins and may be consulted for further information (41, 57, 59, 78, 101, 102, 113, 120, 121, 122, 123, 127, 128, 129).

## Conclusion

In less than a year, there are now more than 100 directly proteomic-relevant articles already published in peer-reviewed journals. In this short period, scientific and medical research progress on the COVID-19 pandemic virus, SARS-CoV-2, has been prodigious, also representing a testament to the (funding of) decades worth of basic research required for such rapid progress when confronting new challenges. While the constraints of this review have prevented a comprehensive presentation of all relevant literature (and led to the exclusion of several topics such as the use of native MS in pharmacological studies), each subsection will hopefully facilitate the readers ability to further investigate specific SARS-CoV-2–related topics of interest. From basic proteomics and potential diagnostics through to a deeper understanding of host-wide changes that occur during infection that could inform potential therapeutic strategies, it is clear that the contribution of the proteomics community to addressing the COVID-19 pandemic is already substantial. Future research will continue to build on existing findings and is highly likely to generate novel insight into the COVID-19 pandemic.

## Supplemental data

This article contains supplemental data (130, 131, 132, 133, 134, 135, 136, 137).

## Conflict of interest

The authors declare no competing interests.
